# Genome Sequence of *Lactobacillus delbrueckii* subsp. *lactis* CNRZ327, a Dairy Bacterium with Anti-Inflammatory Properties

**DOI:** 10.1128/genomeA.00328-14

**Published:** 2014-07-17

**Authors:** Hela El Kafsi, Johan Binesse, Valentin Loux, Julien Buratti, Samira Boudebbouze, Rozenn Dervyn, Amal Hammani, Emmanuelle Maguin, Maarten van de Guchte

**Affiliations:** a INRA, UMR1319 Micalis, Jouy en Josas, France; b AgroParisTech, UMR Micalis, Jouy en Josas, France; c INRA, UR1077 Mathématique Informatique et Génome, Jouy en Josas, France

## Abstract

*Lactobacillus delbrueckii* subsp. *lactis* CNRZ327 is a dairy bacterium with anti-inflammatory properties both *in vitro* and *in vivo*. Here, we report the genome sequence of this bacterium, which appears to contain no less than 215 insertion sequence (IS) elements, an exceptionally high number regarding the small genome size of the strain.

## GENOME ANNOUNCEMENT

*Lactobacillus delbrueckii* is a member of the acidophilus complex, a group of lactobacilli related to *Lactobacillus acidophilus*. This group comprises a relatively high number of bacteria with alleged probiotic properties, particularly in immune modulation, mostly isolated from the human gut environment (*L. acidophilus*, *Lactobacillus johnsonii*, and *Lactobacillus gasseri*). *L. delbrueckii*, rather known from its dairy applications in yogurt and cheesemaking, is an atypical member of this group (1, 2) characterized by a genomic G+C content of about 50%, as opposed to about 35% for the aforementioned bacteria, and its immune modulation potential has often been ignored. We recently identified *L. delbrueckii* subsp. *lactis* CNRZ327 as a strain with strong immune modulation potential capable of downregulating nuclear factor kappa B (NF-κB) activation in gut epithelial HT29 cells *in vitro* and improving the *in vivo* symptoms of dextran sodium sulfate (DSS)-induced colitis in mice, a model of human ulcerative colitis (3, 4).

Here, we report the genome sequence of *L. delbrueckii* subsp. *lactis* CNRZ327, which was determined to near completion. A draft sequence was generated by 454 paired-end sequencing (Roche Life Sciences), followed by sequence assembly using Newbler 2.6 (Roche). The resulting 33 scaffolds were ordered using Mauve aligner (5), with the earlier published genome sequence of *L. delbrueckii* subsp. *bulgaricus* ATCC 11842 (2) as the reference. For most scaffolds, this order was confirmed using PCR amplification of the scaffold-linking sequences, while the position and orientation of the remaining scaffolds were determined using multiplex PCR (6). Thirty-three scaffolds and 33 PCR products together formed a circular chromosome. PCR product size and end-sequences typically converged to indicate the presence of an rRNA operon or an IS element (identified by a transposase-coding sequence) between the scaffolds, in which case no further sequencing of the PCR product was undertaken. Only if the product size indicated the presence of additional and possibly nonrepeated sequences was the PCR product sequenced in order to obtain a complete overview of chromosomally encoded functions. The same approach was used to analyze the 128 gaps between contigs, within the scaffolds, of which 127 appeared to contain IS elements. A total of 1,938,538 bp of chromosomal sequence was assembled, and the total size of the remaining gaps (containing repeated sequences) was estimated at 167 kbp, yielding a total genome size of approximately 2.1 Mbp. Sequence annotation was performed using the AGMIAL annotation platform (7), and protein localization was predicted using SurfG+ (8).

The *L. delbrueckii* subsp. *lactis* CNRZ327 genome appears to contain no less than 215 IS elements, an exceptionally high number regarding the small genome size of the strain (9, 10) and compared to the 56 IS elements for the earlier reported genome of the *L. delbrueckii* subsp. *bulgaricus* strain ATCC 11842 (2). In spite of this abundance of IS elements, which might have resulted in massive gene inactivation and genomic rearrangements, strain CNRZ327 appears to have more extensive metabolic capacities than strain ATCC 11842, and the genomes of the two strains appear to be essentially colinear.

### Nucleotide sequence accession number.

The genome sequence of *L. delbrueckii* subsp. *lactis* CNRZ327 has been deposited in the European Nucleotide Archive (http://www.ebi.ac.uk/ena) under accession no. CCDV01000001.
